# Dietary sodium modulates nephropathy in *Nedd4-2*-deficient mice

**DOI:** 10.1038/s41418-019-0468-5

**Published:** 2019-12-04

**Authors:** Jantina A. Manning, Sonia S. Shah, Tanya L. Henshall, Andrej Nikolic, John Finnie, Sharad Kumar

**Affiliations:** 1 Centre for Cancer Biology, University of South Australia and SA Pathology, Adelaide, SA 5001 Australia; 2 Faculty of Medicine, University of Adelaide, Adelaide, SA 5005 Australia; 3 SA Pathology, Frome Road, Adelaide, SA 5000 Australia

**Keywords:** Ubiquitin ligases, Kidney diseases

## Abstract

Salt homeostasis is maintained by tight control of Na^+^ filtration and reabsorption. In the distal part of the nephron the ubiquitin protein ligase Nedd4-2 regulates membrane abundance and thus activity of the epithelial Na^+^ channel (ENaC), which is rate-limiting for Na^+^ reabsorption. *Nedd4-2* deficiency in mouse results in elevated ENaC and nephropathy, however the contribution of dietary salt to this has not been characterized. In this study we show that high dietary Na^+^ exacerbated kidney injury in *Nedd4-2*-deficient mice, significantly perturbing normal postnatal nephrogenesis and resulting in multifocal areas of renal dysplasia, increased markers of kidney injury and a decline in renal function. In control mice, high dietary Na^+^ resulted in reduced levels of ENaC. However, *Nedd4-2*-deficient kidneys maintained elevated ENaC even after high dietary Na^+^, suggesting that the inability to efficiently downregulate ENaC is responsible for the salt-sensitivity of disease. Importantly, low dietary Na^+^ significantly ameliorated nephropathy in *Nedd4-2*-deficient mice. Our results demonstrate that due to dysregulation of ENaC, kidney injury in *Nedd4-2*-deficient mice is sensitive to dietary Na^+^, which may have implications in the management of disease in patients with kidney disease.

## Introduction

Early stages of chronic kidney disease (CKD), such as histological changes to the glomerular, tubular, interstitial and/or vascular compartments result in the destruction of the renal parenchyma and functional nephron loss [1], as well as fibrosis and inflammation [2]. Early onset CKD can be attributed to monogenic mutations in one of ≥200 genes [3]. Recently, *Nedd4-2* has been identified as a novel gene that protects against early onset CKD, with mice deficient for *Nedd4-2* displaying many features of early stage CKD pathology [4]. In addition, decreased expression of *NEDD4L* (human *Nedd4-2*) has been correlated with early stage human diabetic nephropathy [5].

Despite recent advances in identifying genetic causes of nephropathy, the mechanisms that connect underlying genetic defects with environmental factors associated with the progression of kidney disease, such as high dietary salt intake are poorly understood [6]. The regulation of Na^+^ homeostasis in response to dietary salt requires complex coordination between renal tubular filtration and reabsorption [7]. Reabsorption of Na^+^ is mediated by multiple channels and transporters along the length of the nephron including the epithelial Na^+^ channel (ENaC) and the Na^+^Cl^−^ co-transporter (NCC) [8]. In the late distal convoluted tubule, the connecting tubule and the collecting duct, ENaC is the rate-limiting factor in Na^+^ reabsorption.

ENaC is a heterotrimeric channel composed of three subunits; α, β, and γ, which undergoes complex processing and activation [9]. Short inhibitory peptides in the α and γ subunits are cleaved by proteases to release the active channel [10–12], and N-linked glycan remodeling on all subunits is required for folding, expression’ and function [10]. Many factors contribute to the regulation of ENaC, including inhibition by extracellular Na^+^ [13]. In mice, dietary salt has been reported to modulate ENaC subunit expression [14–16], as high dietary Na^+^ decreases cell surface abundance of ENaC whereas low dietary Na^+^ increases cell surface abundance [16]. Similarly, in salt-resistant Sprague-Dawley rats, increased dietary Na^+^ reduces ENaC, resulting in a decrease in ENaC-driven reabsorption of Na^+^ [14, 17]. Conversely, low dietary Na^+^ activates ENaC [18]. However, in Dahl salt-sensitive rats, increased dietary Na^+^ leads to the hyperactivation of ENaC [17, 19]. Further support for the role of ENaC in modulating Na^+^ reabsorption from dietary salt intake is demonstrated in patients with Liddle syndrome [20] and related mouse models [4, 21], where elevated ENaC function is associated with salt-sensitive hypertension.

The amount of ENaC on the membrane is a primary determinant of its function, with ubiquitination a major pathway controlling the retrieval of this channel from the cell surface [8]. Nedd4-2, a HECT domain containing ubiquitin ligase, binds to PY motifs within the C-termini of all three ENaC subunits to facilitate removal from the membrane [22–24]. Deletion or mutations in PY motifs are known to be associated with increased ENaC and hypertension in Liddle patients [20]. Increased ENaC levels and activity are also seen in *Nedd4-2*-deficient mice, highlighting the relevance of this regulation *in vivo* [25]. Previously, we demonstrated that elevated ENaC contributes to the development of kidney disease in *Nedd4-2*-deficient mice, as treatment with the ENaC inhibitor amiloride ameliorated the observed nephropathy [4, 26].

Here, we show for the first time that improper regulation of ENaC in the absence of Nedd4-2 in mice is exacerbated under conditions of high dietary Na^+^, resulting in nephropathy that is strikingly sensitive to dietary Na^+^. Importantly, a low Na^+^ diet dramatically reduces kidney damage. As the function of Nedd4-2 in regulating Na^+^ homeostasis is highly conserved in mouse and man, our findings are potentially important for the management and treatment of nephropathy in human patients.

## Methods

### Study approval and mouse lines

All animal studies were approved by the institutional ethics and biosafety committees of SA Pathology/CALHN/University of South Australia and were carried out according to the National Health and Medical Research Council of Australia guidelines. *Nedd4-2*^*−/−*^ mice and kidney-specific *Nedd4-2*-deficient mice (*Nedd4-2*^*Ksp1.3*^*)* were generated in our laboratory previously [4, 27] and bred at the SA Pathology animal care facility or UniSA Core Animal Facility (Adelaide, Australia) under specific pathogen free conditions.

### Variable salt feeding and sample collection

*Nedd4-2*^*+/−*^ females were time mated with *Nedd4-2*^*+/*−^ males. Upon the observation of a vaginal plug, diet was changed to low sodium chow (0.05% Na^+^), standard sodium chow (0.2% Na^+^), or high sodium chow (3.1% Na^+^) (Specialty Feeds, WA, Australia). Sample sizes were determined according to G Power calculations and approved by the Institutional Ethics Committee, and randomly assigned to treatment groups, non-blinded due to the nature of disease progression. For embryonic studies, pregnant *Nedd4-2*^*+/−*^ mothers (five for each diet condition) were humanely killed by cervical dislocation and embryos taken at embryonic day (E) 18.5. Embryos were removed from the yolk sac, decapitated, and tail tip was taken for genotyping. Kidneys were dissected, and one placed into Histochoice reagent (ProSciTech, Kirwan, QLD, Australia) for the histological analysis of paraffin embedded or frozen samples. For paraffin samples, kidneys were transferred to 70% ethanol and then embedded in paraffin. Kidneys for frozen sectioning were soaked in 30% sucrose overnight before being embedded in OCT (ProSciTech, Kirwan, QLD, Australia). The remaining kidney was snap frozen in liquid nitrogen for immunoblot and mRNA analysis. For the *Nedd4-2*^*Ksp1.3*^ strain, low and standard Na^+^ diet was continued during pregnancy and lactation, and in solid chow of male and female pups until they were humanely killed for analysis at 40 days. High-Na^+^ diet was continued during pregnancy and lactation until the pups were humanely killed for analysis at 20 days. At the time of collection, mice were anaesthetized, blood collected by cardiac puncture, and organs dissected after cervical dislocation. The capsule was removed, and one kidney was snap frozen in liquid nitrogen, the other was cut in half in the coronal plane and immersion fixed in Histochoice for 48 h at 4 °C. One half of the kidney was paraffin embedded and the other OCT embedded as above. Nine mice of each genotype, for each diet condition were analyzed.

### Histological analysis

Sections (5 μm) were cut using a paraffin microtome, de-paraffinized with xylene, and dehydrated through a graded series of ethanol. Slides were stained with hematoxylin-eosin using standard protocols. To evaluate collagen deposition using picrosirius red, slides were stained for 1 h in saturated picric acid with 0.1% Direct Red 80 (Sigma-Aldrich), then washed in acidified water for 2 min. Digital images were acquired by using a NanoZoomer (Hamamatsu).

### Immunostaining

Immunostaining for KIM-1 and all ENaC subunits were carried out on frozen sections (14 μm). Tissue sections were blocked with 10% goat serum and incubated with primary antibodies: rat anti-KIM-1 (cat. # MAB1817, R&D systems); rabbit anti-α-ENaC and rabbit anti-γ-ENaC [28]; rabbit anti-β-ENaC [27], or rabbit anti-NCC (cat. No. ab3553; Abcam). Sections were then incubated with the corresponding fluorescently tagged secondary antibody (AlexaFluor-488, Thermo Fisher Scientific), counterstained with DAPI, and mounted in Prolong Gold Antifade reagent (Invitrogen). Stained samples were imaged using an LSM 800 confocal microscope using Zen 2011 (Black Edition) version 8.1.5.484 (Carl Zeiss Microscopy, Jena, Germany). Image analysis was conducted using Adobe image suite software.

### Immunoblotting

Half of each kidney was lysed in ice-cold extraction buffer at pH 7.5 (50 mM Tris-HCl pH 7.5, 1 mM EDTA, 1 mM EGTA, 0.27 M sucrose, 0.1% β-mercaptoethanol, and HALT protease and phosphatase inhibitor cocktail [Thermo Fisher Scientific]). Tissue was homogenized, frozen in liquid nitrogen, immediately thawed, and incubated at 4 °C on a Nutator for 30 min and centrifuged at 13,000 rpm for 5 min. Supernatant protein (25 μg) was combined with protein load buffer (100 mM Tris-HCl pH 6.8, 200 mM DTT, 4% SDS, 0.2% bromophenol blue, and 20% glycerol), heated at 37 °C for 30 min, loaded onto 4–20% precast SDS-PAGE gels (Bio-Rad), and transferred to PVDF membrane using the Trans-blot Turbo instrument (Bio-Rad). Membranes were blocked with 5% skim milk in TBS-T (Tris-buffered saline/0.05% Tween 20) and primary antibodies added; anti-α, β or γ-ENaC, anti-NCC (as described above), anti-Nedd4-2 [4], and mouse anti-β-actin (clone AC15; Sigma-Aldrich). For ENaC, NCC, and Nedd4-2 antibodies, HRP secondary antibodies (Millipore) were added and developed with West Femto (Thermo Scientific). β-actin was developed using Cy5 secondary (GE Healthcare). Images were acquired on a ChemiDoc Touch Imager (Bio-Rad). Quantitation was conducted using Image Lab Software (Bio-Rad), with each band normalised to β-actin and presented as fold change from control standard Na^+^ condition.

### Real-time quantitative PCR

Total RNA was isolated from half of each kidney using TRIzol Reagent (Life Technologies) and RNA was reverse-transcribed with a high capacity cDNA reverse transcription kit (Applied Biosciences). qRT-PCR was performed and analyzed as described [29], where all data are normalized to TBP (TATA-box binding protein) levels. Primer sequences are detailed below:

**Table:** 

Gene	Primer sequence (5′–3′)
Collagen-1 (*Col1a1*)	F: CGGAGAAGAAGGAAAACGAGGAG
	R: CACCATCAGCACCAGGGAAAC
Vimentin (*Vim*)	F: CGGCTGCGAGAGAAATTGC
	R: CCACTTTCCGTTCAAGGTCAAG
Kidney injury molecule 1 (*KIM-1*)	F: TGGTTGCCTTCCGTGTCTCT
	R: TCAGCTCGGGAATGCACAA
TATA-box binding protein (*TBP*)	F: CAAACCCAGAATTGTTCTCCTT
	R: ATGTGGTCTTCCTGAATCCCT

### Electrolytes and aldosterone

Electrolytes, aldosterone, and other parameters of kidney function in blood were measured at SA Pathology. Plasma Na^+^, K^+^, Cl^−^, Ca^2+^, and creatinine were measured using an Advia 2400 chemistry system (Siemens), and urine osmolality with an Advanced 3320 osmometer (Advanced Instruments). Plasma aldosterone levels were measured using a Liaison Aldosterone Kit and a Chemiluminescent Analyzer (Diasorin, Saluggia, Italy).

### Statistical analysis of data

Statistical analysis was performed using GraphPad Prism software (v6.0). A Mann–Whitney test for nonparametric data was used to assess changes in blood parameters. All other data were analysed using unpaired 2-tailed Student’s *t* test. A *P* value of ≤0.05 was considered significant. All values are presented as mean ± SEM.

## Results

### High dietary Na^+^ exacerbates kidney injury

**Fig. 1:**
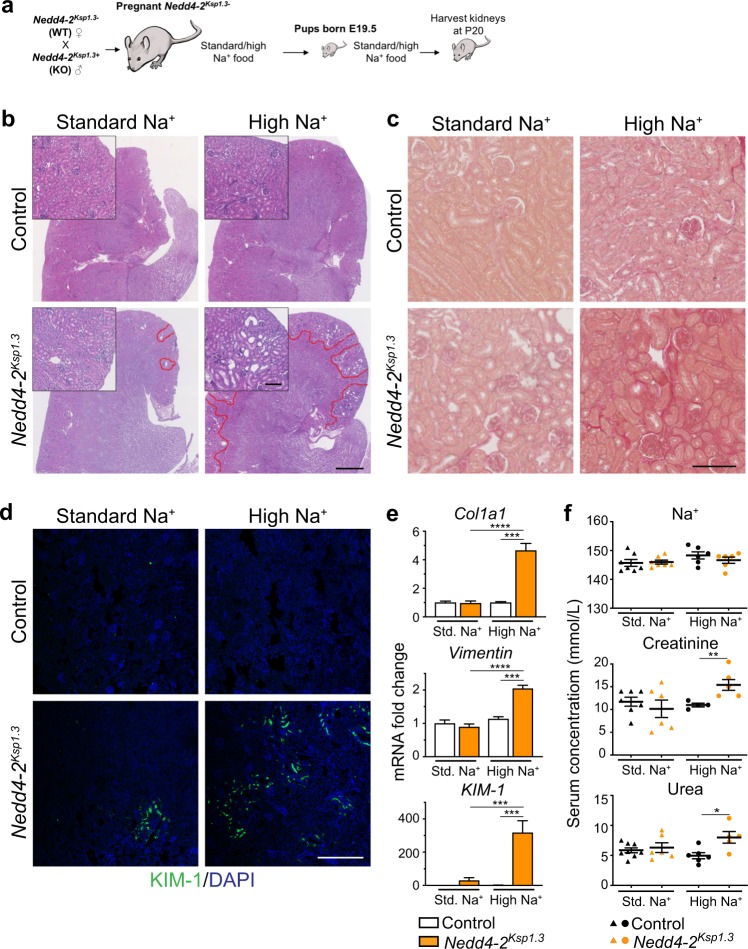
High dietary Na^+^ exacerbates kidney pathology in *Nedd4-2*^*Ksp1.3*^ mice. **a** Outline of salt-feeding experiments. **b** Representative H&E images show increase in kidney injury after high-Na^+^ diet in P20 kidneys. Red lines denote areas of damage. Scale bar: 1 mm, inset 100 µm. **c** Picrosirius red staining shows fibrosis after the high-Na^+^ diet in *Nedd4-2*^*Ksp1.3*^ kidneys. Scale bar: 100 µm. **d** KIM-1 staining shows small regions of injury in *Nedd4-2*^*Ksp1.3*^ kidneys under standard-Na^+^ diet, and extensive regions after high-Na^+^ diet. Scale bar: 250 µm. **e** qPCR for markers of kidney injury *collagen I* (*Col1a1), vimentin* and *KIM-1* show the significant increases in *Nedd4-2*^*Ksp1.3*^ kidneys after the high-Na^+^ diet (*n* = 6–7 mice per genotype). Data are shown as fold change from control on standard (Std.) diet, mean ± SEM with significance calculated by a Student’s *t* test (two tailed). **f** Serum levels of Na^+^ remain unchanged, however, creatinine and urea are increased in *Nedd4-2*^*Ksp1.3*^ mice after the high-Na^+^ diet. Data are presented as one point per mouse, mean ± SEM with significance calculated by a Mann–Whitney test. *P < 0.05, **P < 0.01, *P < 0.005, **P < 0.001.

Mice deficient for *Nedd4-2* progressively develop kidney disease accompanied by higher expression of ENaC and NCC, resulting in increased Na^+^ reabsorption [4]. To investigate whether high dietary Na^+^ would induce earlier onset of disease, or an exacerbated disease phenotype, we utilized renal tubule specific *Nedd4-2*-deficient mice, *Nedd4-2*^*Ksp1.3*^. Pregnant mice were fed a standard Na^+^ (0.2%) or high-Na^+^ (3.1%) diet for the duration of pregnancy/lactation and continued in the solid food of pups (Fig. 1a). Initially, we found that this diet caused lethality of the first high Na^+^ fed *Nedd4-2*^*Ksp1.3*^ mouse at P38. Hence, subsequent mice on high-Na^+^ diet were analyzed at postnatal day (P) 20, since we have previously shown the initiation of pathology at this age [4]. On a standard Na^+^ diet, kidneys of *Nedd4-2*^*Ksp1.3*^ mice were similar to control mice, with only minor pathology comprised of a few small, focal areas of dysplasia in the cortex (Fig. 1b). After a high-Na^+^ diet, there were numerous multifocal areas of renal dysplasia, which were characterized by cortical immaturity in the form of small, hypercellular glomeruli with inapparent capillaries, immature tubules (with often dilated lumina, which sometimes contained desquamated, degenerate epithelial lining debris), and persistent interstitial mesenchyme (Fig. 1b and Supplementary Fig. 1). Many collecting ducts were also immature and sometimes disorganized. Picrosirius red staining revealed a small increase in fibrosis in *Nedd4-2*-deficient kidneys (Fig. 1c) and KIM-1 immunohistochemistry demonstrated more extensive regions of renal damage (Fig. 1d). Markers of renal injury; *collagen*, *vimentin,* and *KIM-1* were all significantly increased in *Nedd4-2*^*Ksp1.3*^ kidneys after 20 days of the high-Na^+^ diet (Fig. 1e).

The renal tubule specific *Nedd4-2*^*Ksp1.3*^ mice do not present any signs of kidney pathology until P20 and have a normal lifespan. However, a model of complete knockout of *Nedd4-2* is predominantly perinatal lethal, with the few surviving pups displaying some evidence of kidney pathology soon after birth (from ~3 days of age) [27]. Heterozygous *Nedd4-2* mice have a normal lifespan [27]. We therefore investigated whether high dietary Na^+^ fed to pregnant *Nedd4-2*^*+/*−^ mice would induce earlier onset of the disease during fetal development of *Nedd4-2*^−*/−*^ pups (Supplementary Fig. 2a). Varied dietary Na^+^ in pregnancy has been previously reported to affect the fetus, indicating the transfer of maternal Na^+^ to the embryonic environment [30, 31]. No evidence of kidney pathology or fibrosis was observed in *Nedd4-2*^*−*^^*/−*^ fetuses just prior to birth at E18.5 after the standard or high-Na^+^ diet (Supplementary Fig. 2b–d), indicating that a high-Na^+^ diet during pregnancy does not induce fetal kidney damage in mice lacking *Nedd4-2*. Therefore, our results show that high-Na^+^ levels exacerbate kidney damage caused by the lack of Nedd4-2, postnatally.

### High dietary Na^+^ impairs kidney function in *Nedd4-2*^*Ksp1.3*^ mice

The analysis of serum from mice fed either standard or high-Na^+^ diet for 20 days showed that levels of Na^+^ or K^+^ remain unaltered (Fig. 1f and Table 1). Interestingly, Cl^−^ levels were significantly reduced in the *Nedd4-2*^*Ksp1.3*^ mice after a high-Na^+^ diet, suggesting some Na^+^ retention or fluid loss such as in AQP-2-deficient mice [32] (Table 1). Due to the small size of the animals, metabolic studies to measure urine volume and glomerular filtration rate were not feasible in these mice. Importantly, despite other parameters remaining similar, creatinine and urea levels were significantly increased in *Nedd4-2*^*Ksp1.3*^ mice after the high-Na^+^ diet, suggesting that kidney function is compromised (Fig. 1f and Table 1).

**Table 1:** P20 serum analysis of Control and *Nedd4-2*^*Ksp1.3*^ mice.

	Standard Na^+^		High Na^+^	
	Control (*n* = 7)	*Nedd4-2*^*Ksp1.3*^ (*n* = 6–7)	Control (*n* = 4–6)	*Nedd4-2*^*Ksp1.3*^ (*n* = 5–6)
K^+^ (mM)	3.63 ± 0.21	3.93 ± 0.20	4.50 ± 0.26	3.53 ± 0.65
Cl^−^ (mM)	111.40 ± 0.65	110.10 ± 0.96	**112.00** ± 1.21^**^	**102.02** ± 1.96^**^
Ca^2+^ (mM)	2.63 ± 0.03	2.49 ± 0.08	2.55 ± 0.1	2.61 ± 0.05
Protein (g/L)	40.57 ± 1.09	40.86 ± 1.86	41.17 ± 1.78	45.00 ± 0.63
HCO_3_^−^ (mM)	18.86 ± 1.7	18.00 ± 0.49	18.17 ± 1.66	22.20 ± 1.86
Anion gap (mM)	19.14 ± 0.99	21.71 ± 0.64	23.60 ± 2.25	24.60 ± 2.64
Albumin (g/L)	12.86 ± 0.26	12.71 ± 0.57	12.50 ± 0.77	14.20 ± 0.49
Globulin (g/L)	27.71 ± 0.89	28.14 ± 1.32	28.67 ± 1.02	30.60 ± 0.68

Data presented as mean ± SEM for number of mice (*n*) indicated in parentheses. Significance was determined using a Mann–Whitney test for non-normally distributed data

**P < 0.01, comparing bold values

### Low dietary Na^+^ ameliorates kidney injury in *Nedd4-2*^*Ksp1.3*^ mice

**Fig. 2:**
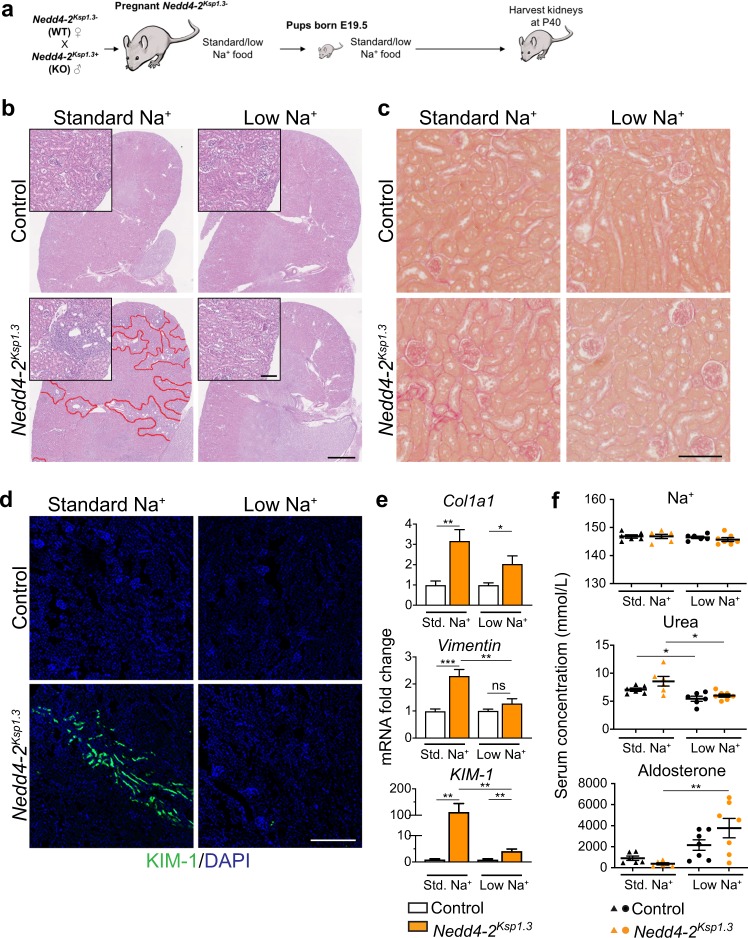
Low dietary-Na^+^ ameliorates kidney pathology in *Nedd4-2*^*Ksp1.3*^ mice. **a** Outline of salt-feeding experiments. **b** Representative H&E images show decreased injury in *Nedd4-2*^*Ksp1.3*^ mice after low-Na^+^ diet in P40 kidneys. Red lines denote areas of damage. Scale bar: 1 mm, inset 100 μm. **c** Picrosirius red staining shows a decrease in fibrosis after the low-Na^+^ diet. Scale bar: 100 μm. **d** KIM-1 staining reveals some regions of kidney injury in *Nedd4-2*^*Ksp1.3*^ kidneys under standard-Na^+^ diet, but no detectable regions after low-Na^+^ diet. Scale bar: 250 μm. **e** qPCR for markers of kidney injury, *vimentin* and *KIM-1* show a significant reduction in *Nedd4-2*^*Ksp1.3*^ kidneys after the low-Na^+^ diet. *Vimentin* levels are comparable to control kidneys on a low-Na^+^ diet (*n* = 6–7 mice per genotype). Data are shown as fold change from control on standard (Std.) diet, mean ± SEM with significance calculated by a Student’s *t* test (two tailed). **f** Serum levels of Na^+^ remain unchanged, however, the low-Na^+^ diet causes a decrease in urea in both control and *Nedd4-2*^*Ksp1.3*^ mice. Aldosterone is increased in the *Nedd4-2*^*Ksp1.3*^ mice after the low-Na^+^ diet. Data are presented as one point per mouse, mean ± SEM with significance calculated by a Mann–Whitney test. *P < 0.05, **P < 0.01, *P < 0.005.

We next assessed whether a low Na^+^ (0.05%) diet could ameliorate or reduce kidney damage (Fig. 2a). As described previously, areas of kidney damage were obvious in *Nedd4-2*^*Ksp1.3*^ kidneys on a standard Na^+^ diet at P40 [4], however after a low Na^+^ diet no kidney damage was apparent (Fig. 2b). Following the low Na^+^ feeding there was a reduction in fibrosis in *Nedd4-2*^*Ksp1.3*^ kidneys compared with the standard Na^+^ diet, as demonstrated by picrosirius red staining for collagen (Fig. 2c). Furthermore, KIM-1 staining revealed a reduction in regions of kidney injury when compared with the standard Na^+^ diet (Fig. 2d). *Collagen*, *vimentin*, and *KIM-1* mRNA levels were all significantly increased in *Nedd4-2*^*Ksp1.3*^ mice compared with controls on a standard Na^+^ diet (Fig. 2e). After the low Na^+^ diet, levels of *vimentin* and *KIM-1* were significantly reduced in the *Nedd4-2*^*Ksp1.3*^ kidneys. In addition, *vimentin* levels were now comparable to control animals on the low Na^+^ diet. Together these data indicate that a low Na^+^ diet ameliorates kidney pathology in *Nedd4-2*^*Ksp1.3*^ mice.

### Low dietary Na^+^ alters blood parameters in *Nedd4-2*^*Ksp1.3*^ mice

The analysis of plasma electrolytes revealed no changes between control and *Nedd4-2*^*Ksp1.3*^ animals on a standard Na^+^ diet at P40 (Fig. 2f and Table 2). In addition, electrolyte levels were not altered by the low Na^+^ diet in either group, however urea was decreased in both control and *Nedd4-2*^*Ksp1.3*^ animals (Fig. 2f). There was also a significant increase in aldosterone levels in *Nedd4-2*^*Ksp1.3*^ animals in response to the low Na^+^ diet, suggesting an imbalance in the hormonal control of electrolytes. Furthermore, there was a trend toward increased total protein, anion gap and globulin levels in the *Nedd4-2*^*Ksp1.3*^ animals, which were all significantly reduced after the low Na^+^ diet, indicating some improvement of kidney function (Table 2).

**Table 2:** P40 serum analysis of Control and *Nedd4-2*^*Ksp1.3*^ mice.

	Standard Na^+^		Low Na^+^	
	Control (*n* = 6)	*Nedd4-2*^*Ksp1.3*^ (*n* = 5–6)	Control (*n* = 6–7)	*Nedd4-2*^*Ksp1.3*^ (*n* = 7)
K^+^ (mM)	4.17 ± 0.22	4.10 ± 0.20	4.23 ± 0.13	4.37 ± 0.22
Cl^−^ (mM)	109.50 ± 0.22	108.30 ± 1.02	110.00 ± 0.68	110.00 ± 0.38
Ca^2+^ (mM)	2.46 ± 0.04	2.46 ± 0.04	2.45 ± 0.03	2.37 ± 0.04
Creatinine (uM)	17.17 ± 0.65	18.67 ± 1.23	16.29 ± 1.55	15.43 ± 1.13
Protein (g/L)	46.67 ± 0.49	**48.00** ± 0.58^**^	45.17 ± 1.22	**44.86** ± 0.34^**^
HCO_3_^−^ (mM)	21.00 ± 0.73	19.67 ± 0.88	21.67 ± 0.76	21.57 ± 0.75
Anion gap (mM)	20.33 ± 0.71	**23.20** ± 0.73^*^	19.17 ± 1.30	**18.43** ± 1.13^*^
Albumin (g/L)	14.83 ± 0.31	15.00 ± 0.37	14.17 ± 0.48	13.86 ± 0.34
Globulin (g/L)	31.83 ± 0.48	**33.00** ± 0.58^*^	31.00 ± 0.97	**31.00** ± 0.31^*^

Data presented as mean ± SEM for number of mice (*n*) indicated in parentheses. Significance was determined using a Mann–Whitney test for non-normally distributed data

*P *<* 0.05, **P *<* *0.01* , comparing values in bold

### ENaC levels are altered in response to high dietary Na^+^

**Fig. 3:**
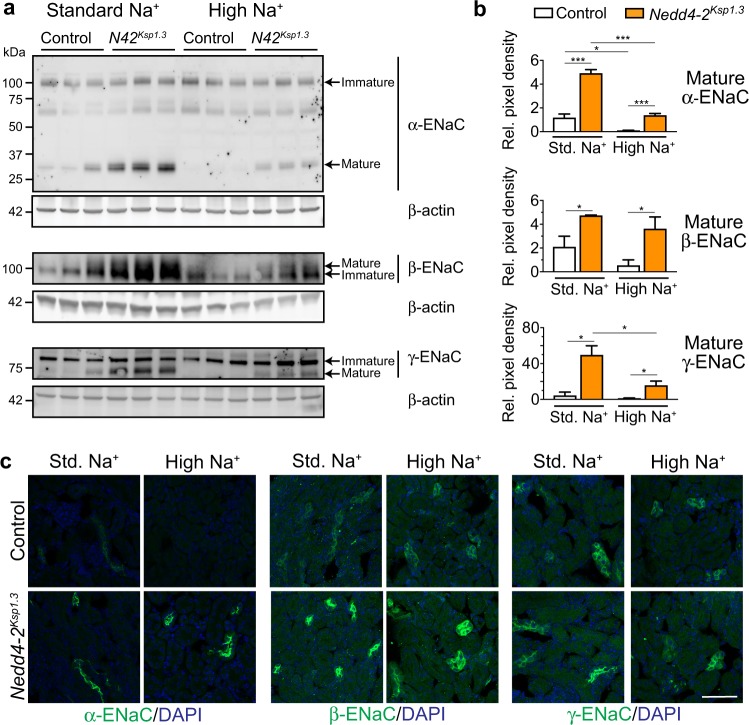
Downregulation of ENaC levels after high-Na^+^ diet is compromised in *Nedd4-2*^*Ksp1.3*^ kidneys. **a** Semi-quantitative immunoblot analysis reveals decreased expression of mature ENaC in control and *Nedd4-2*^*Ksp1.3*^ (*N42*^*Ksp1.3*^) kidneys after a high-Na^+^ diet, however, levels remain elevated in *Nedd4-2*^*Ksp1.3*^ kidneys. Expression levels quantitated in **b** as fold change from control standard diet, relative to β-actin expression. **c** Immunohistochemical staining of ENaC subunits reveals increased membrane localisation in *Nedd4-2*^*Ksp1.3*^ kidneys. High Na^+^ results in reduced membrane α-ENaC in control kidneys, but not in *Nedd4-2*^*Ksp1.3*^ kidneys. Scale bar: 50 μm. *n* = 3, mean ± SEM with significance calculated by a Student’s t test (2 tailed). *P < 0.05, ***P < 0.005.

Nedd4-2 is well-known to regulate ENaC [25], but the effect of dietary Na^+^ intake on this regulation and its contribution to the salt-sensitivity of disease has not been characterized. Hence, we sought to analyze ENaC levels in response to dietary Na^+^ in control and *Nedd4-2*-deficient mice. Full length ENaC undergoes processing into active forms; present as cleaved products for α-ENaC of ~30 kDa and γ-ENaC predominantly as ~70 kDa [12, 33–35]. The mature form of β-ENaC is evident as a band with slower mobility by immunoblot due to glycan remodeling [10]. At P20, *Nedd4-2*^*Ksp1.3*^ mice showed a significant increase in the mature forms of all ENaC subunits when compared with control mice (Fig. 3a and quantitated in Fig. 3b). The presence of a band at ~65 kDa for α-ENaC may represent an alternative cleavage product or a nonspecific band detected by this antibody. In response to the high-Na^+^ diet, control animals showed a significant decrease in cleaved mature α-ENaC levels, and a trend toward decreased mature β and γ-ENaC (Fig. 3b). Mature forms of α and γ-ENaC were significantly decreased in *Nedd4-2*^*Ksp1.3*^ kidneys after the high-Na^+^ diet. Importantly, these levels still remained higher than control animals. These mature forms of ENaC are predominantly localized at the cell membrane to form the active channel [36]. Immunostaining of membrane localized ENaC subunits confirmed the increased expression of ENaC in *Nedd4-2*^*Ksp1.3*^ kidneys under a standard Na^+^ diet (Fig. 3c). After the high-Na^+^ diet, a decrease in the α subunit was observed in control animals, however membrane levels of all three subunits remained high in *Nedd4-2*^*Ksp1.3*^ kidneys. This correlated with an increase in Nedd4-2 protein expression (Supplementary Fig. 3a and b), similar to that in a previous study where dietary salt was reported to modulate Nedd4-2 [37].

At the fetal level, *Nedd4-2*^*−/*−^ kidneys displayed increased levels of the mature forms of all three ENaC subunits when mothers were fed a standard, low or high-Na^+^ diet during pregnancy (Supplementary Fig. 4a and b), confirming that Nedd4-2 can regulate ENaC *in utero*. However, compared with the standard Na^+^ diet fed groups, both control and *Nedd4-2*^*−/−*^ animals did not show changes in the expression of any of the mature forms or membrane localized ENaC after a high-Na^+^ diet (Supplementary Fig. 4). Therefore, the loss of Nedd4-2 postnatally impeded the ability to reduce ENaC levels in response to a high-Na^+^ diet.

### ENaC levels are increased in response to low dietary Na^+^

**Fig. 4:**
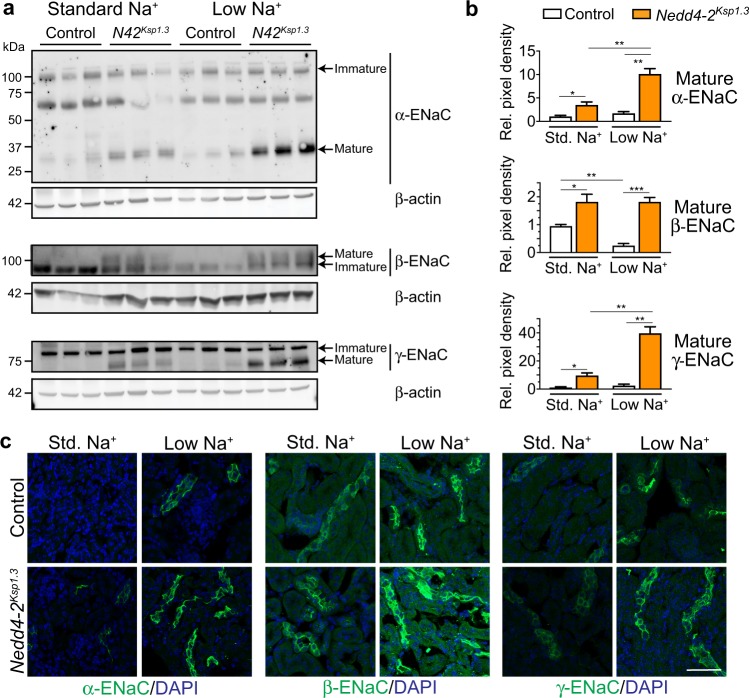
ENaC levels are increased in response to low dietary Na^+^. **a** Semi-quantitative immunoblot analysis reveals an increased expression of mature ENaC subunits in *Nedd4-2*^*Ksp1.3*^ (*N42*^*Ksp1.3*^) kidneys. A low-Na^+^ diet causes an increase in α and γ-ENaC in *Nedd4-2*^*Ksp1.3*^ kidneys only. Expression levels quantitated in **b** as fold change from control standard diet, relative to β-actin expression. **c** Immunohistochemical staining of ENaC subunits reveals increased membrane localization of all subunits in *Nedd4-2*^*Ksp1.3*^ kidneys. Low dietary Na^+^ results in the upregulation of membrane staining of all three ENaC subunits in control and *Nedd4-2*^*Ksp1.3*^ kidneys. Scale bar: 50 μm. *n* = 3, mean ± SEM with significance calculated by a Student’s *t* test (2 tailed). *P < 0.05, ***P < 0.01, ***P < 0.005.

We next assessed whether low dietary Na^+^ was affecting ENaC levels in *Nedd4-2*^*Ksp1.3*^ mice at P40, when nephropathy becomes more advanced. In control animals, levels of the mature cleaved forms of α and γ-ENaC remained similar after a standard or low Na^+^ diet and mature β-ENaC was expressed at very low levels (Fig. 4a and quantitated in Fig. 4b). However, in the *Nedd4-2*^*Ksp1.3*^ mice, α and γ-ENaC were significantly increased after the low Na^+^ diet. Immunostaining of kidney sections indicated higher levels of all three subunits of ENaC localized on the membrane of tubules in *Nedd4-2*^*Ksp1.3*^ mice compared with controls (Fig. 4c). Low Na^+^ diet increased ENaC abundance in both control and *Nedd4-2*^*Ksp1.3*^ mice, without affecting Nedd4-2 levels (Fig. 4c and Supplementary Fig. 3c and d).

In fetal kidneys, low Na^+^ caused a significant increase in cleaved mature γ-ENaC products in both control and *Nedd4-2*^−*/*−^ animals, although an increase in membrane localized ENaC subunits was not apparent (Supplementary Fig. 4). These results demonstrate that a low Na^+^ diet postnatally increases mature ENaC expression, augmenting the amount of Na^+^ that can be reabsorbed by the kidneys.

### NCC is regulated by dietary Na^+^ in control and *Nedd4-2*^*Ksp1.3*^ mice

**Fig. 5:**
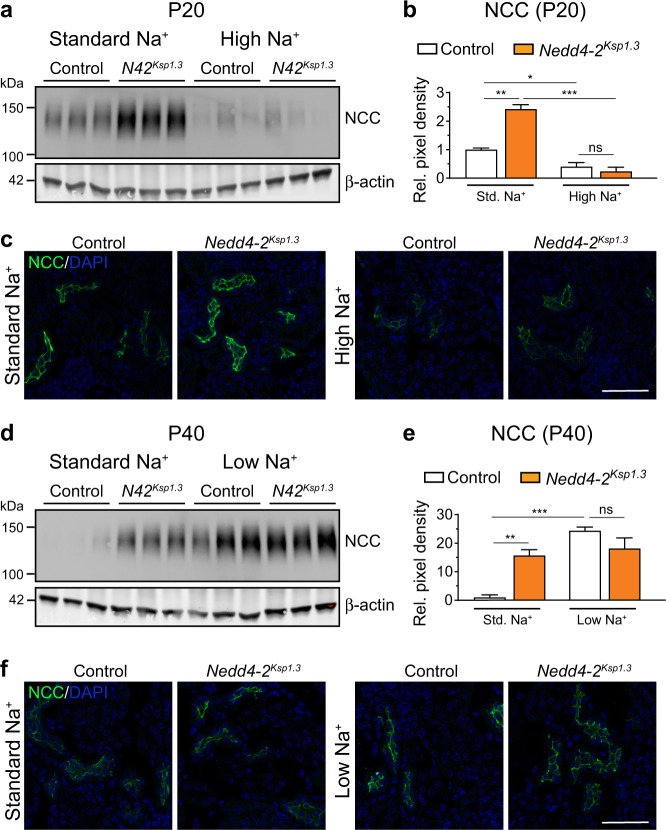
Changes in NCC levels in response to varied Na^+^ diet. **a** Semi-quantitative immunoblot analysis of P20 kidneys shows higher expression of NCC in *Nedd4-2*^*Ksp1.3*^ (*N42*^*Ksp1.3*^) kidneys under standard-Na^+^ diets, downregulated in both control and *Nedd4-2*^*Ksp1.3*^ kidneys after high-Na^+^ diet. Expression levels quantitated in **b** as fold change from control standard diet, relative to β-actin expression. **c** NCC has higher membrane expression in *Nedd4-2*^*Ksp1.3*^ kidneys at P20 on standard-Na^+^ diet, downregulated to a similar level in control and *Nedd4-2*^*Ksp1.3*^ kidneys after high-Na^+^ diet. **d** Semi-quantitative immunoblot analysis of P40 kidneys shows higher expression of NCC in *Nedd4-2*^*Ksp1.3*^ kidneys under standard-Na^+^ diet, upregulated in control and *Nedd4-2*^*Ksp1.3*^ kidneys after low-Na^+^ diet. Expression levels quantitated in **e** as fold change from control standard diet, relative to β-actin expression. **f** Higher membrane expression of NCC in *Nedd4-2*^*Ksp1.3*^ kidneys at P40 on standard-Na^+^ diet, upregulated to a similar level in control and *Nedd4-2*^*Ksp1.3*^ kidneys after low-Na^+^ diet. For graphs, *n* = 3, mean ± SEM with significance calculated by a Student’s t test (2 tailed). *P < 0.05, **P < 0.01, ***P < 0.005. Scale bar: 50 μm.

In addition to ENaC, NCC also mediates Na^+^ reabsorption in the distal convoluted tubule and has been shown to be regulated by Nedd4-2 [4, 38]. In support of this, *Nedd4-2*^*Ksp1.3*^ mice in our study showed increased NCC at P20. Following the high-Na^+^ diet, both control and *Nedd4-2*^*Ksp1.3*^ kidneys downregulated total NCC to a similar extent (Fig. 5a, b). Furthermore, the membrane localization of NCC was decreased in both genotypes (Fig. 5c), suggesting that NCC is unlikely to be responsible for the high-Na^+^ exacerbated kidney damage. At P40, *Nedd4-2*^*Ksp1.3*^ mice again had higher expression of total NCC (Fig. 5d and e) and membrane localized NCC (Fig. 5f). A low Na^+^ diet resulted in a significant increase in total and membrane localized NCC in control kidneys such that no difference was observed between the genotypes after this diet (Fig. 5d–f). Together, results from this study suggest that ENaC is the primary contributor to the salt-sensitivity of kidney disease in *Nedd4-2*^*Ksp1.3*^ mice.

## Discussion

*Nedd4-2* deficiency leads to a progressive nephropathy that is associated with increased levels of functional membrane associated ENaC [4]. We now demonstrate that nephropathy in *Nedd4-2*^*Ksp1.3*^ mice is highly dependent on dietary salt, as kidney pathology is exacerbated by high-Na^+^ and ameliorated by low Na^+^. Our data support that this is largely driven by increased ENaC in these mice, as another Na^+^ transporter ubiquitinated by Nedd4-2, NCC [39], is still downregulated in *Nedd4-2*^*Ksp1.3*^ mice in response to high Na^+^. Further, the presence of renal dysplasia at later stages postnatally suggests that Nedd4-2 is likely to be required for normal nephron development after birth, as well as for protection against high-Na^+^ induced renal damage.

High salt intake and the corresponding changes in Na^+^ and K^+^ transport are known to cause renal damage [40–42]. Previous studies have demonstrated that varied dietary salt fed in pregnancy is able to pass through to embryos and offspring [30, 31]. We found that a high-Na^+^ diet fed to pregnant *Nedd4-2*^*+/−*^ mice did not cause any apparent kidney pathology in *Nedd4-2*^*−/*−^ fetuses by E18.5, suggesting that *Nedd4-2*^*−/*−^ mice can maintain sodium homeostasis during embryonic development. This diet fed to pregnant renal tubule specific *Nedd4-2*^*Ksp1.3*^ mice and continued during lactation led to renal dysplasia and exacerbated kidney injury by P20, indicating that the kidney disease is salt-sensitive during postnatal stages. The specific contribution of high dietary Na^+^ in pregnancy, lactation, the solid food of pups or a combination of these stages, remains to be explored. In contrast, a low Na^+^ diet suppressed the development of the disease, such that renal injury was barely detectable by P40. Elevated Na^+^ intake can lead to hypertension, resulting in subsequent renal damage [42, 43]. High dietary Na^+^ has also been reported to induce tubular injury in the absence of elevated blood pressure, particularly in rats [42, 44]. As we were unable to measure blood pressure in this study due to the small size of mice at P20 and P40, further studies are required to determine the contribution of hypertension, if any, to the salt-sensitivity of kidney disease in *Nedd4-2*^*Ksp1.3*^ mice.

Mature forms of all three ENaC subunits were increased in fetuses and young mice lacking *Nedd4-2*. High-Na^+^ diet fed to pregnant mothers did not affect ENaC levels in either wild-type or *Nedd4-2*^*−/*−^ fetuses. Postnatally, a high-Na^+^ diet resulted in lower levels of α-ENaC in wild-type mice, correlating with the increased Nedd4-2 levels, in support of a previous study where Nedd4-2 levels were shown to be regulated by dietary Na^+^ [37]. *Nedd4-2*-deficient mice, which have elevated membrane associated ENaC subunits on a standard laboratory diet, failed to efficiently downregulate α-ENaC after the high-Na^+^ feeding, similar to Dahl salt-sensitive rats [45]. We propose that the high levels of ENaC aid in the reabsorption of elevated Na^+^ which in turn exacerbates kidney damage in *Nedd4-2*^*Ksp1.3*^ mice. Hypertonicity caused by high NaCl concentrations is known to alter cellular responses [46]. Various mechanisms have been proposed to explain how high Na^+^ induces renal tubular damage, such as via apoptosis, glomerular hyperfiltration, activation of the renin-angiotensin-aldosterone system, oxidative stress and proteinuria [42, 47]. Understanding the direct contribution of elevated ENaC to the damaged renal parenchyma caused by high dietary Na^+^ remains to be established.

Conversely, a low Na^+^ diet increased membrane localized mature ENaC expression. Despite this, kidney pathology was ameliorated in *Nedd4-2*^*Ksp1.3*^ mice fed on this diet. Hence, we hypothesize that despite high ENaC levels, the low amount of Na^+^ in the diet is sufficient to limit disease progression.

Increased expression and membrane localization of ENaC subunits has been observed in other models of *Nedd4-2* deficiency, contributing to the observed phenotypes [4, 21, 25, 27, 48, 49]. A model of inducible renal-specific *Nedd4-2* knockout in proximal and distal tubules, and collecting ducts (*Nedd4L*^*Pax8/LC1*^) revealed a hypertensive phenotype only after a high-Na^+^ diet, attributed to the increased levels of NCC [38]. Interestingly, in our study NCC levels were downregulated to a similar level in both wild-type and *Nedd4-2*^*Ksp1.3*^ kidneys, suggesting that this is unlikely to be responsible for the salt-sensitivity of nephropathy. *Nedd4L*^*Pax8/LC1*^ mice displayed increased β and γ-ENaC after a high-Na^+^ diet when compared with control mice. The absence of kidney injury in these mice may be explained by a lack of increased mature α-ENaC or the specific location or timing of Nedd4-2 knockout in this inducible system [25].

*Nedd4-2* (*NEDD4L*) variants and single nucleotide polymorphisms (SNPs) are known to be associated with human hypertension [50] and end-stage renal disease due to autosomal dominant polycystic kidney disease and juvenile nephronopthisis [51]. The effect of these SNPs on ENaC levels in these patients has not been investigated. *NEDD4L* variants in patients with CKD-like pathologies have not yet been characterized, however decreased expression of *NEDD4L* has recently been observed in early diabetic nephropathy [5]. This supports our findings that loss of *NEDD4L* may contribute to the development of nephropathy, with the potential for its expression levels to be utilized as a biomarker for early stage disease. It has been reported that short term salt reduction in people with CKD reduced blood pressure and proteinuria [52], however the effects of Na^+^ restriction on primary endpoints and progression to end-stage renal disease have not been assessed. Findings in this study warrant future endeavors into providing robust clinical evidence for a need to restrict dietary Na^+^ in patients with potential susceptibility to kidney disease.

## Supplementary information

Supplemental Material
