# Supplementary material for: Dietary sodium modulates nephropathy in Nedd4-2-deficient mice
Source: Cell Death Differ. 2019 Dec 4;27(6):1832–43. doi: 10.1038/s41418-019-0468-5 (PMC7244563; doi:10.1038/s41418-019-0468-5)

Supplementary Information

This document contains Supplementary Figure Legends and 4 supplementary figures (PDFs):

Supplementary Figure Legends

Supplementary Fig. 1. H&E histology of *Nedd4-2*$^{Ksp1.3}$ kidney at P20 after high Na^+ diet.

Supplementary Fig. 2: High dietary Na^+ does not affect nephrogenesis in *Nedd4-2*$^{-/-}$ mice.

Supplementary Fig. 3: Changes in Nedd4-2 levels in response to varied Na^+ diet.

Supplementary Fig. 4: Changes in fetal ENaC levels in response to varied Na^+ diet.

Supplementary Figure Legends

Supplementary Fig. 1. H&E histology of *Nedd4-2$^{Ksp1.}$3* kidney at P20 after high Na^+ diet.

Multifocal cortical areas of dysplasia with immature glomeruli (arrows) and dilated tubules (*). Intervening areas of cortex are of normal morphology. Scale bar: 100 µm.

Supplementary Fig. 2: High dietary Na^+ does not affect nephrogenesis in *Nedd4-2$^{-/-}$* mice

a Outline of salt feeding experiments. **b** Representative H&E images show no evidence of pathology in standard or high Na^+ fed E18.5 kidneys, with inset showing higher magnification. Scale bar: 0.5 mm, inset 100 µm. **c** Picrosirius red staining reveals no fibrosis after either diet. Scale bar: 100 µm. **d** qPCR for markers of kidney injury *collagen I* (*Col1a1*), *vimentin* and *KIM-1* show no significant changes in mRNA levels (n = 4 mice per genotype). Data are shown as fold change from control on standard (Std.) diet, mean + SEM with significance calculated by a Student's t test (2 tailed).

Supplementary Fig. 3: Changes in Nedd4-2 levels in response to varied Na^+ diet.

a Semi-quantitative immunoblot analysis reveals higher expression of Nedd4-2 (doublet showing two isoforms) in control and *Nedd4-2$^{Ksp1.3}$* P20 kidneys after high Na^+ diet. As Nedd4-2 is only ablated in cells expressing Ksp in the *Nedd4-2$^{Ksp1.3}$* line, remaining Nedd4-2 bands in *Nedd4-2$^{Ksp1.3}$* samples are due to expression of this protein in regions of the kidney not driven by the Ksp promoter. **b** Levels from (a) quantitated as fold change from control standard diet, relative to β-actin expression. **c** Semi-quantitative immunoblot analysis reveals no change in Nedd4-2 expression in control and *Nedd4-2$^{Ksp1.3}$* P40 kidneys after low Na^+ diet. **d** Levels from (c) quantitated as fold change from control standard diet, relative to

β-actin expression. n = 3, mean + SEM with significance calculated by a Student's t test (2 tailed). *P<0.05.

Supplementary Fig. 4: Changes in fetal ENaC levels in response to varied Na^+ diet.

a Semi-quantitative immunoblot analysis reveals higher expression of mature ENaC subunits in *Nedd4-2*$^{-/-}$ kidneys at E18.5, under standard, high and low Na^+ diets. Expression levels quantitated in **b** as fold change from control standard diet, relative to β-actin expression. **c** Immunohistochemical staining of ENaC subunits reveals increased membrane localization in *Nedd4-2*$^{-/-}$ mice. Scale bar: 50 µm. n = 3 (except for high Na^+ control where n = 2), mean + SEM with significance calculated by a Student's t test (2 tailed). *P<0.05, **P<0.01, ***P<0.005.

Supplementary Fig. 1.

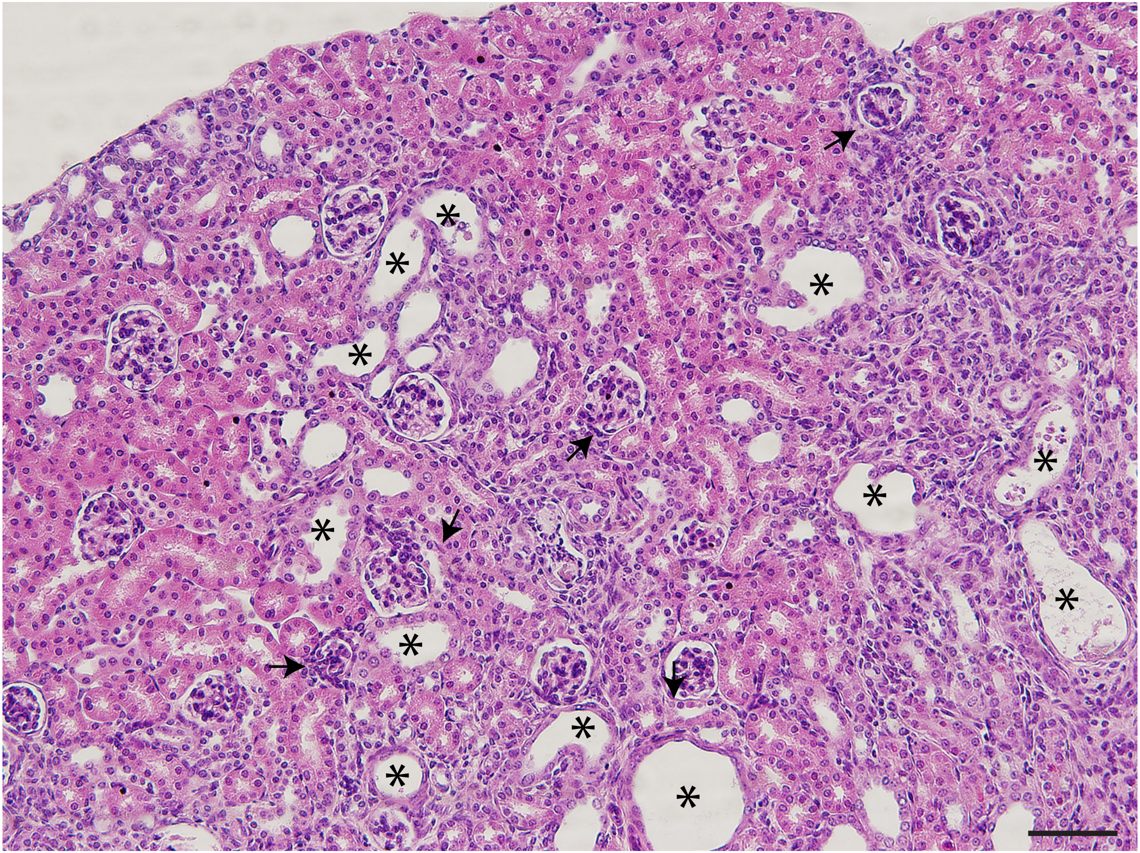

Supplementary Fig. 2.

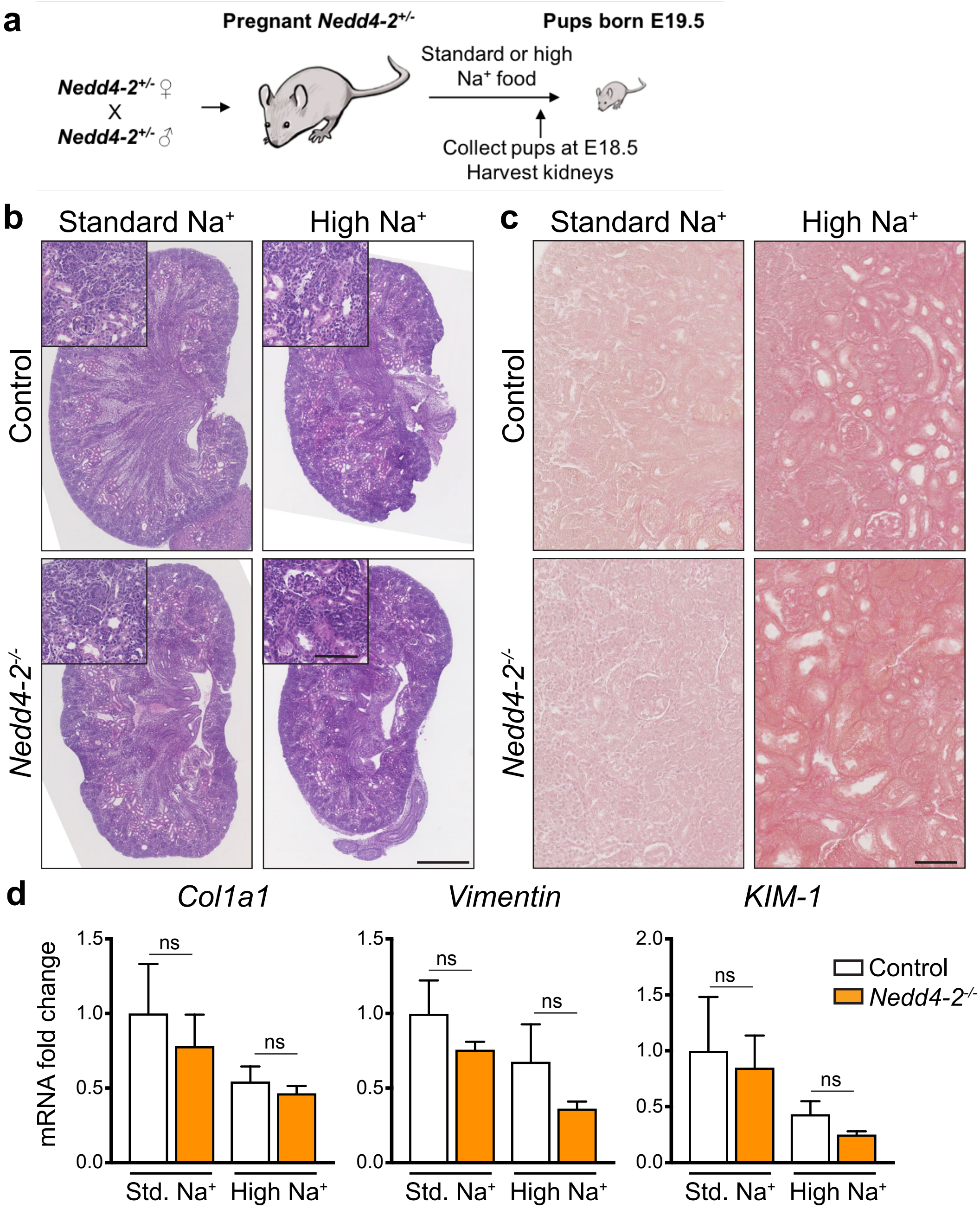

Supplementary Fig. 3.

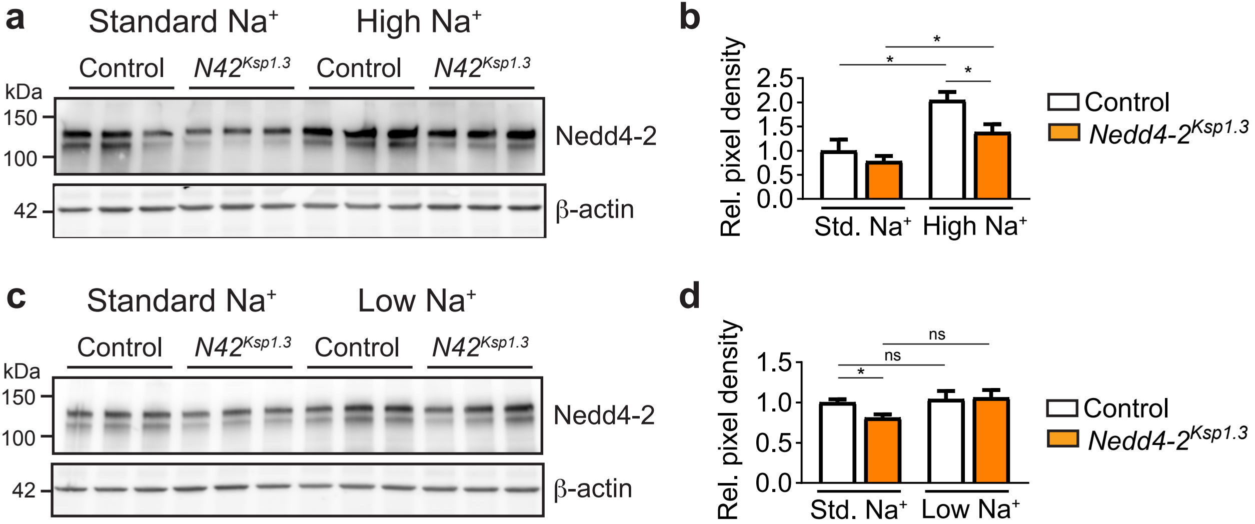

Supplementary Fig. 4.

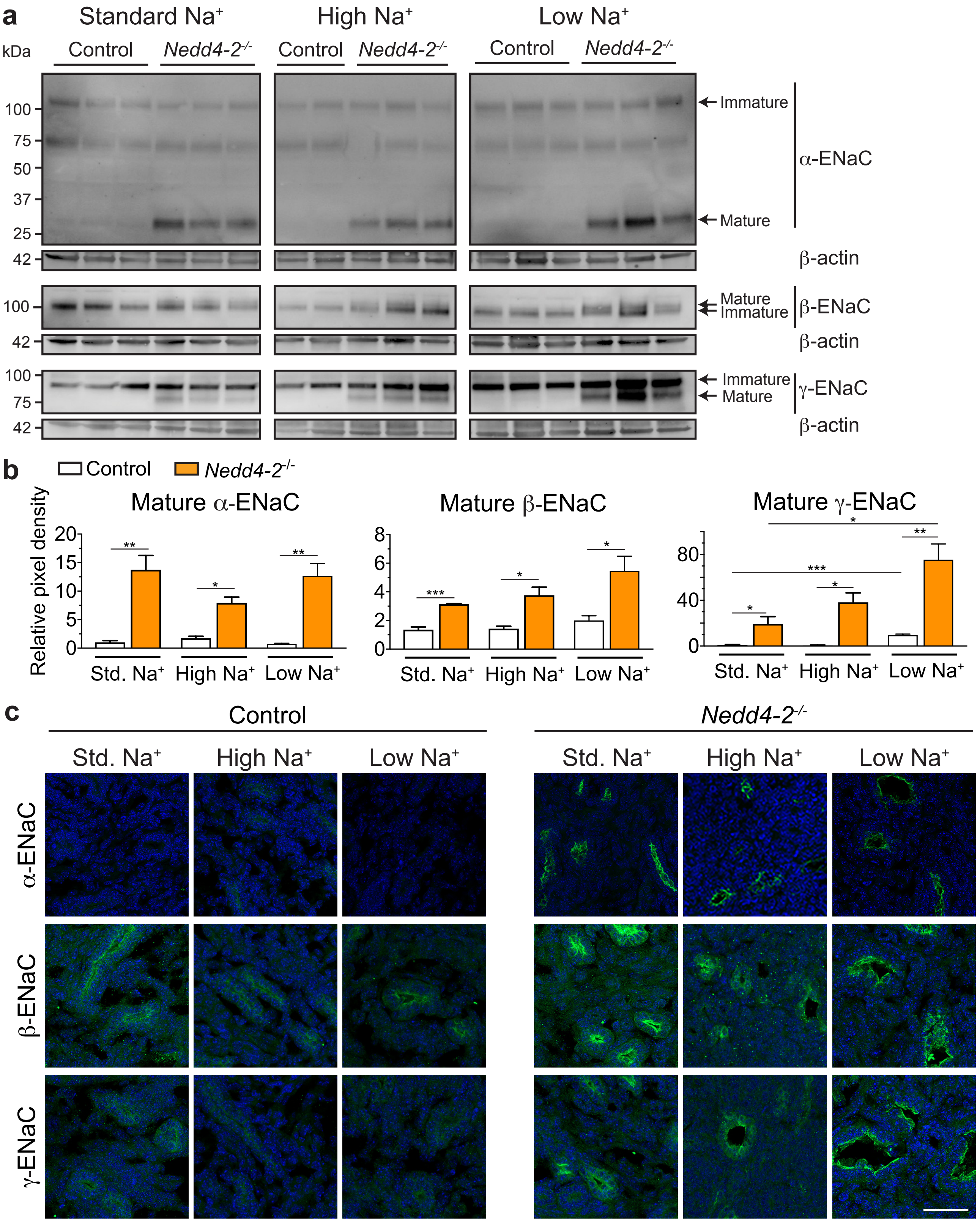

Supplement: Supplementary file 1 — Supplemental Material [file 41418_2019_468_MOESM1_ESM.pdf]
